# Rapid Identification and Susceptibility Testing of *Candida* spp. from Positive Blood Cultures by Combination of Direct MALDI-TOF Mass Spectrometry and Direct Inoculation of Vitek 2

**DOI:** 10.1371/journal.pone.0114834

**Published:** 2014-12-09

**Authors:** Evgeny A. Idelevich, Camilla M. Grunewald, Jörg Wüllenweber, Karsten Becker

**Affiliations:** Institute of Medical Microbiology, University Hospital Münster, Münster, Germany; Institute of Microbiology, Switzerland

## Abstract

Fungaemia is associated with high mortality rates and early appropriate antifungal therapy is essential for patient management. However, classical diagnostic workflow takes up to several days due to the slow growth of yeasts. Therefore, an approach for direct species identification and direct antifungal susceptibility testing (AFST) without prior time-consuming sub-culturing of yeasts from positive blood cultures (BCs) is urgently needed. Yeast cell pellets prepared using Sepsityper kit were used for direct identification by MALDI-TOF mass spectrometry (MS) and for direct inoculation of Vitek 2 AST-YS07 card for AFST. For comparison, MALDI-TOF MS and Vitek 2 testing were performed from yeast subculture. A total of twenty four positive BCs including twelve *C. glabrata*, nine *C. albicans*, two *C. dubliniensis* and one *C. krusei* isolate were processed. Applying modified thresholds for species identification (score ≥1.5 with two identical consecutive propositions), 62.5% of BCs were identified by direct MALDI-TOF MS. AFST results were generated for 72.7% of BCs directly tested by Vitek 2 and for 100% of standardized suspensions from 24 h cultures. Thus, AFST comparison was possible for 70 isolate-antifungal combinations. Essential agreement (minimum inhibitory concentration difference ≤1 double dilution step) was 88.6%. Very major errors (VMEs) (false-susceptibility), major errors (false-resistance) and minor errors (false categorization involving intermediate result) amounted to 33.3% (of resistant isolates), 1.9% (of susceptible isolates) and 1.4% providing 90.0% categorical agreement. All VMEs were due to fluconazole or voriconazole. This direct method saved on average 23.5 h for identification and 15.1 h for AFST, compared to routine procedures. However, performance for azole susceptibility testing was suboptimal and testing from subculture remains indispensable to validate the direct finding.

## Introduction

Advanced treatment strategies in oncology, transplantation and other fields of medicine led to a considerable increase in the number of immunocompromised patients during the past decades [1]. As a consequence, opportunistic infections, such as invasive candidiasis, are increasingly observed [2]. *Candida* spp. is among the four most common isolates in nosocomial bloodstream infections (BSI) [3]. Sepsis caused by *Candida* spp. has higher mortality than that due to bacterial pathogens, reaching 53.7%–63.5% in *Candida* associated septic shock [4], [5]. Adequate source control and antifungal therapy administered within 24 hours of shock is crucial in these patients, since mortality rate rises to 97.6% in patients who don’t attain these goals [5]. However, early administration of appropriate antifungal therapy is challenging for many reasons. First, antifungals are not included in most empiric therapy regimens [6] and swift microbiological detection of fungi as a causing agent is highly important to initiate antifungals. This still does not guarantee that initiated antifungal therapy is adequate. To some extent, susceptibilities to antifungals for given species can be predicted based on local epidemiological susceptibility data [2]. However, fungi can hardly be recognized on species level from Gram stain of positive blood culture (BC), as demonstrated in a recent study [7]. In this setting, species prediction by Gram stain is further hampered by the current increase in non-*albicans* species among *Candida* causing BSIs [8], [9]. Furthermore, even if species is known, it became difficult to reliably infer susceptibility of a given isolate since resistance increase is currently observed in many *Candida* species, including occurrence of multidrug resistant yeasts [10], [11]. Several novel technologies have been suggested to rapidly identify yeasts directly from positive BCs, including PNA-FISH [7] and procedures based on matrix-assisted laser desorption ionization–time of flight mass spectrometry (MALDI-TOF MS) [7], . However, antifungal susceptibility testing (AFST) still needs sub-cultured isolates, which are then subjected to different AFST methods. This classical workflow takes up to several days starting from the blood sampling due to the slow growth of yeasts [16], thus, resulting in a timeframe that is not consistent with the concept of rapid microbiological diagnostics. An approach of using very short-term cultures incubated only few hours after streaking of positive BC broth onto solid medium has recently been suggested for identification of BSI pathogens by MALDI-TOF [18]. This approach can conveniently be combined with an early inoculation of an automated susceptibility testing device from the same biomass, thus contributing to an earlier identification and antimicrobial susceptibility testing result compared to standard methods [19]. Such method is very useful for bacteria, but poorly performable for yeasts due to their slow growth.

In this study, we therefore investigated a combined approach of rapid identification and susceptibility testing of *Candida* spp. from positive BCs by simultaneous direct MALDI-TOF MS and direct inoculation of Vitek 2 from yeast cell pellets without prior time-consuming sub-culturing.

## Materials and Methods

### Study design

Positive monomicrobial BCs containing yeasts as detected by Gram stain were prospectively included into the study from February to September 2013. All types of BC bottles (BACTEC Plus Aerobic/F, BACTEC Plus Anaerobic/F, BACTEC Mycosis-IC/F and BACTEC Peds Plus/F, BD Diagnostics, Heidelberg, Germany) incubated in an automated BC system (BACTEC 9240, BD Diagnostics) were included. If the samples could not be investigated immediately after positive signal, they were kept for study activities in refrigerator for maximum one day. A preliminary experiment has demonstrated that the cell number in positive BC bottles only minimally changes if bottles are kept at +5.5°C for up to three days compared to a considerable CFU increase at room temperature (data not shown). The median time from yeast detection by Gram stain of a positive BC broth to the processing according to the study protocol was 4.8 h (range 19 min to 24.2 h). All routine diagnostics of positive BCs were performed immediately.

### Direct identification by MALDI-TOF MS using Sepsityper kit

Direct identification from positive BCs was performed using Sepsityper kit (Bruker Daltonics, Bremen, Germany) including ethanol/formic acid protein extraction (E/FA) as previously described [14]. Briefly, 1 ml of BC broth was placed into a reaction tube followed by lysis of blood cells, centrifugation and wash steps. The resulting yeast cell pellet was dissolved in deionized water and subjected to ethanol followed by centrifugation. Subsequent drying of yeast cell pellet for 10 min tended to provide better identification scores compared to 0, 20, or 30 min in a preliminary experiment (data not shown), and was therefore used throughout the study. After drying, usually small pellet was suspended in the adjusted volume of 10 µl 70% formic acid and equal volume of acetonitrile and again centrifuged. 1 µl of supernatant was pipetted onto target slides in triplicate, dried and overlaid with HCCA matrix solution for MALDI-TOF MS analysis. The spectra were acquired by the Microflex LT system (Bruker Daltonics) with the FlexControl automatic mode and analysed using MALDI Biotyper 3.0 software (Bruker Daltonics). Criterion of successful species identification was defined as score value ≥1.5 on at least one spot provided that the first two proposed results in the list were identical. However, it was tried to achieve score ≥1.7 by applying additional procedures, if this score value was not reached directly by the standard protocol described above. First, additional shot series each of 40 shots were manually triggered on five different positions within a spot. If ≥1.7 score was still not achieved, it was tried to use a double layer of extracted protein (a total of 2 µl of supernatant), which was again subjected to an automatic measurement, and additional manual shots as described above, if the score ≥1.7 was not achieved (S1 Figure).

For comparison, MALDI-TOF MS was performed from yeast subculture on Kimmig agar after 24 h incubation using tube-based E/FA extraction as previously described [20] as standard method, and additionally on intact yeast cells by direct transfer of colony material without E/FA extraction.

### Direct susceptibility testing by direct inoculation of yeast cell pellet prepared using Sepsityper kit into Vitek 2

A yeast cell pellet was prepared using Sepsityper kit as described above. The pellet was dissolved in 0.45% saline solution to prepare a suspension with McFarland turbidity 1.8 to 2.2 recommended by Vitek 2 (bioMérieux, Marcy l’Etoile, France) manufacturer for susceptibility testing of yeasts [21]. Finally, yeast suspension was directly inoculated into Vitek 2 susceptibility testing device using AST-YS07 cards (bioMérieux, Marcy l’Etoile, France). The AFST results were available for amphotericin B, fluconazole, voriconazole, caspofungin and flucytosine. Yeast suspension was inoculated even if only lower values than recommended McFarland turbidity 1.8 to 2.2 were achieved. However, in these cases, another suspension was prepared from two yeast pellets and tested to investigate effect of inoculum on AFST results. For comparison, AFST was performed by inoculation of a standard 1.8 to 2.2 McFarland suspension prepared from yeast subculture after 24 h incubation on Kimmig agar. This control testing was performed simultaneously in triplicate to minimize the impact of reproducibility errors on comparison between direct AFST and standard 24 h testing. Median values for minimum inhibitory concentrations (MICs) were calculated and served as reference values. MIC values were used to assign isolates into susceptible (S), intermediate (I), or resistant (R) category, based on the Vitek 2 breakpoint setting “EUCAST 2013+ CLSI 2013 D” applied in the routine diagnostics at the time of the study (amphotericin B S≤1, R≥2; fluconazole S≤2, I = 4, R≥8 except for *C. glabrata* [S≤1, 2≤I≤32, R≥64]; voriconazole S≤0.125, R≥0.25; caspofungin S≤2; flucytosine S≤4, 8≤I≤16, R≥32). Very major error (VME, number of false susceptible results of direct AFST divided by the number of isolates tested resistant by the standard method), major error (ME, number of false resistant results of direct AFST divided by the number of susceptible isolates as determined by the reference method) and minor error (mE, false categorization involving intermediate result divided by the total number of tested isolates) rates were calculated compared to the standard testing from 24 h cultures, as recommended by the International Organization for Standardization (ISO) [22] and the US Food and Drug Administration (FDA) [23] guidances. Categorical agreement (CA, results within the same category) and essential agreement (EA, minimum inhibitory concentration difference ≤1 double dilution step) were determined. Additionally, MIC_50_ and MIC_90_ for tested antifungals were calculated and compared between direct and standard method.

### Vital cell count

Colony forming units (CFU) counting was performed on positive BC broths, on suspensions prepared from cell pellets for direct AFST, and on standardized 1.8 to 2.2 McFarland suspensions from 24 h sub-cultures for standard testing. Six 1∶10 dilutions of respective fluids were plated onto Kimmig agar, followed by colony counting after 24 h incubation and CFU determination of correspondent inocula.

### Statistical analysis

Statistical analysis was performed using the Mann-Whitney-Wilcoxon test, or the Wilcoxon signed-rank test, as appropriate (GraphPad Prism 5.0, GraphPad Software, Inc., La Jolla, CA, USA). Statistical significance was assumed for p-values <0.05.

## Results

A total of 24 processed positive monomicrobial BCs from 15 patients included 13 aerobic, 5 anaerobic, 5 fungal and 1 paediatric bottle. Overall distribution of yeast species was as follows: *C. glabrata* 50.0% (12/24), *C. albicans* 37.5% (9/24), *C. dubliniensis* 8.3% (2/24) and *C. krusei* 4.2% (1/24). *C. albicans* was detected in 46.7% (7/15), *C. glabrata* in 40% (6/15), *C. dubliniensis* in 6.7% (1/15) and *C. krusei* in 6.7% (1/15) of patients.

### Direct MALDI-TOF identification results

Species identification was achieved in 62.5% (15/24) of samples. All species identification results were correct as compared to the results of the standard method. Four samples had scores ≥2.0, six samples ≥1.7–<2.0 and five samples ≥1.5–<1.7. In 50% (12/24) of samples, species identification was reached directly with standard MALDI Biotyper measurement protocol with following scores: four samples ≥2.0, five samples ≥1.7–<2.0 and three samples ≥1.5–<1.7. Additionally, one sample reached score of ≥1.5–<1.7 after manual shots, one sample was assigned a higher category of ≥1.7–<2.0 after applying a double layer onto the spot, and two samples reached score of ≥1.5–<1.7 after manual shots of a double layer. The species distribution of identified samples and the identification rates by the direct method for different species are presented in Table 1. Direct method provided identification result for 61.5% (8/13), 60% (3/5), 60% (3/5) and 100% (1/1) of aerobic, anaerobic, fungal and paediatric BC bottles, respectively.

**Table 1:** *Candida* species identification rates by using direct MALDI-TOF MS method, n = 24.

Species	Total number ofsamples	Number of identifiedsamples	Identificationrate, %
*C. glabrata*	12	7	58.3
*C. albicans*	9	6	66.7
*C. dubliniensis*	2	1	–a
*C. krusei*	1	1	–a
**Total**	**24**	**15**	**62.5**

a Identification rate was not calculated due to the low number of isolates.

Mean yeast cell count of 15 samples with successful identification by direct MALDI-TOF MS method amounted to 4.5×10^7^ CFU/ml (range 3.0×10^5^ to 3.6×10^8^) compared to 7.3×10^6^ CFU/ml (range 7.5×10^4^ to 2.7×10^7^) of 9 samples with failed identification (p = 0.03).

The mean time needed for direct MALDI-TOF MS method using standard automated measurement was 53.1 min for a single sample and consisted of 6.3 min for lysis-centrifugation steps with Sepsityper kit, 32.2 min for E/FA extraction including 10 min pellet drying time, 11.2 min for spotting of samples onto the target slide and 3.4 min for MALDI-TOF MS measurement. If further processing was needed in case of low scores, it took additional 3.0 min for manual shots, 14.6 min for application of a double layer, 3.0 min for MALDI-TOF MS measurement of a double layer and 3.1 min for manual shots of a double layer. Compared to the standard method identification with E/FA extraction, results were available 23.5 h earlier.

One hundred percent species identification was achieved for control MALDI-TOF MS performed using tube-based E/FA extraction from yeast subculture on Kimmig agar after 24 h incubation. MALDI-TOF MS performed on intact yeast cells by direct transfer of sub-cultured 24 h colony material onto the target slides without E/FA extraction provided species identification in 75.0% (18/24) of samples with scores of ≥2.0 (eight isolates), ≥1.7–<2.0 (nine isolates) and ≥1.5–<1.7 (one isolate). Species identification rates for *C. glabrata* and *C. albicans* using MALDI-TOF MS from sub-cultures without E/FA extraction were 91.7% (11/12) and 66.7% (6/9), respectively. All identification results were correct compared to the identification from 24 h sub-culture with E/FA extraction.

### Direct susceptibility testing using Sepsityper kit and direct inoculation into Vitek 2

Using standard testing of 24 h sub-cultures, results were available for 23 of 24 isolates, due to a general technical device failure in one case. AFST profiles were generated for 100% (23/23) of those isolates. MIC values and categorization were available for all antifungal-isolate combinations except amphotericin B results for two *C. dubliniensis* isolates. Results of standard AFST of 24 h sub-cultures are presented in S1 Table. Results for the direct AFST testing using one cell pellet were available for 22 of 24 isolates, because of a general technical device failure in two cases. Testing of 27.3% (6/22) of isolates was aborted due to insufficient growth in the growth control. Therefore, AFST profiles could be generated for 72.7% (16/22) of tested isolates with direct method. Of these, complete AFST profiles (for all five antifungals tested) were available for 13 isolates. In one isolate, testing for amphotericin B and flucytosine was terminated due to the exceeded incubation time. In two isolates, MIC values were generated only for caspofungin. Thus, comparison of AFST findings between direct and standard methods was possible for 70 antifungal-isolate combinations in total (Table 2). Respective error and agreement rates for performance of direct inoculation method are shown in Table 3.

**Table 2:** Results of antifungal susceptibility testing by direct inoculation method compared to standard Vitek 2 method from 24 h sub-cultures.

Antifungal/species (No. of isolates)	Direct method					Standard method				
	Category			MIC^a^ (µg/ml)		Category			MIC^a^ (µg/ml)	
	S	I	R	50%	90%	S	I	R	50%	90%
**Amphotericin B (13)**	**13**	**0**	**0**	**≤0.25**	**0.5**	**13**	**0**	**0**	**0.5**	**0.5**
* C. albicans* (5)	5	0	0			5	0	0		
* C. glabrata* (7)	7	0	0			7	0	0		
* C. krusei* (1)	1	0	0			1	0	0		
**Fluconazole (14)**	**6**	**0**	**8**	**2**	**4**	**3**	**0**	**11**	**4**	**16**
* C. albicans* (6)	6	0	0			3	0	3		
* C. glabrata* (7)	0	0	7			0	0	7		
* C. krusei* (1)	0	0	1^b^			0	0	1^b^		
**Voriconazole (14)**	**11**	**0**	**3**	**≤0.12**	**0.25**	**10**	**0**	**4**	**≤0.12**	**2**
* C. albicans* (6)	6	0	0			4	0	2		
* C. glabrata* (7)	4	0	3			5	0	2		
* C. krusei* (1)	1	0	0			1	0	0		
**Caspofungin (16)**	**16**	**0**	**0**	**≤0.25**	**≤0.25**	**16**	**0**	**0**	**≤0.25**	**≤0.25**
* C. albicans* (8)	8	0	0			8	0	0		
* C. glabrata* (7)	7	0	0			7	0	0		
* C. krusei* (1)	1	0	0			1	0	0		
**Flucytosine (13)**	**13**	**0**	**0**	**≤1**	**≤1**	**12**	**1**	**0**	**≤1**	**≤1**
* C. albicans* (5)	5	0	0			5	0	0		
* C. glabrata* (7)	7	0	0			7	0	0		
* C. krusei* (1)	1	0	0			0	1	0		

a MIC, minimum inhibitory concentration. MIC_50_ and MIC_90_ were not calculated for single species due to the low number of isolates.

b intrinsic resistant.

**Table 3:** Performance of direct antifungal susceptibility testing compared to the standard method.

Antifungal/species	No. of isolate-antifungalcombinations	Very majorerrors^a^	Majorerrors^a^	Minorerrors^a^	Categoricalagreement	Essentialagreement
**Amphotericin B**	**13**	**0**	**0**	**0**	**100%**	**100%**
* C. albicans*	5					
* C. glabrata*	7					
* C. krusei*	1					
**Fluconazole**	**14**	**3 (27.3%)**	**0**	**0**	**78.6%**	**64.2%**
* C. albicans*	6	3				
* C. glabrata*	7					
* C. krusei*	1					
**Voriconazole**	**14**	**2 (50%)**	**1 (10%)**	**0**	**78.6%**	**85.7%**
* C. albicans*	6	2				
* C. glabrata*	7		1			
* C. krusei*	1					
**Caspofungin**	**16**	**0**	**0**	**0**	**100%**	**100%**
* C. albicans*	8					
* C. glabrata*	7					
* C. krusei*	1					
**Flucytosine**	**13**	**0**	**0**	**1 (7.7%)**	**92.3%**	**92.3%**
* C. albicans*	5					
* C. glabrata*	7					
* C. krusei*	1			1		
**TOTAL**	**70**	**5 (33.3%)**	**1 (1.9%)**	**1 (1.4%)**	**90%**	**88.6%**

a Error rates are calculated according to ISO [22] and FDA [23] guidances. Very major errors (%) - number of false susceptible results of direct AFST divided by the number of isolates tested resistant by the standard method, major errors (%) - number of false resistant results of direct AFST divided by the number of susceptible isolates as determined by the standard method, minor errors (%) - number of false categorizations involving intermediate result divided by the total number of tested isolates.

In 19 isolates, for which recommended McFarland turbidity 1.8 to 2.2 was not achieved with one yeast cell pellet, direct testing was simultaneously performed both using inoculum suspension prepared from one pellet and inoculum suspension from two pellets. Among these 19 isolates, McFarland turbidity 1.8 to 2.2 was achieved with two yeast cell pellets in only three isolates. Comparison of direct AFST performance results for this sub-group (each calculated as compared to the standard method from sub-cultures) demonstrated that there was an improvement of performance, if two pellets were used, due to the lower error rates and higher agreement rates for fluconazole and voriconazole (S2 Table).

Microbial count of 24 suspensions prepared from one cell pellet amounted to a mean value of 2.1×10^6^ CFU/ml (range 0 to 2.8×10^7^) compared to 1.1×10^7^ CFU/ml (range 2.2×10^6^ to 1.9×10^7^) for suspensions of the isolates sub-cultured for 24 h from the same samples (p<0.001). Noteworthy, there was also statistically significant difference between the cell counts of 16 suspensions for which complete AFST profile was generated (mean 2.7×10^6^ CFU/ml, range 0.7×10^3^ to 2.8×10^7^) and six suspensions with aborted measurements due to insufficient growth (mean 1.8×10^3^ CFU/ml, range 0 to 0.7×10^4^), p = 0.002. Yeast cell counts in suspensions prepared from two cell pellets were higher than those prepared from one cell pellet of the same 19 samples: 8.0×10^5^ (range 1.1×10^3^ to 8.0×10^6^) vs. 5.3×10^5^ (range 1.2×10^3^ to 5.3×10^5^), respectively (p<0.01).

The duration of susceptibility testing in Vitek 2 device was 23.9 h in average for directly inoculated samples, compared to 15.1 h for standard inoculation from sub-cultures. 6.3 min were additionally necessary for preparation of cell pellet for direct testing using Sepsityper kit, as described above. However, sub-cultivation of isolates took 24 h prior to standard inoculation. Altogether, the results of direct AFST were available 15.1 h earlier than the results of standard AFST.

## Discussion

Several studies investigated direct identification of yeasts from positive BC vials. These studies used diverse protocols and varied greatly in the achieved results [12]–[17], [24]–[27]. While some authors demonstrated extremely low success rates for fungi [12], [24], others reported excellent identification with different methods of sample processing [13], [17], [27]. In our study with Sepsityper kit, rate of identification to species level was 62.5%. Jamal *et al*. identified 50% and Buchan *et al*. none of yeast positive BCs while both studies used Sepsityper kit [14], [24]. In a study with spiked samples proceeded using Sepsityper kit, 77% of yeast isolates were correctly identified [15]. Yan et al. reported 100% identification by using Sepsityper kit with two additional preprocessing washing steps [17]. In our hands, introduction of these steps did not improve result in a preliminary experiment (data not shown). Noteworthy, we kept positive BC bottles refrigerated if processing had to be delayed, while Yan *et al*. do not provide information on bottles’ storage. During storage at room temperature, increase in yeast cells occurs which might contribute to better direct MALDI-TOF MS identification. Also, no *C. glabrata* isolates were presented among the samples included by Yan *et al.*, which hinders direct comparison between the studies. Our study demonstrated the ability of direct MALDI-TOF MS to identify yeasts from positive BC broth to be dependent on the abundance of yeast cells in a sample, which is in line with data from other investigators [17]. We didn’t identify any effect of BC bottle type on identification results.

Previous studies have shown that the score ≥2.0 recommended by the manufacturer as a criterion for species identification might be too conservative and can be lowered without decrease in accuracy [15], [20], [28]–[31]. We used score ≥1.5 in combination with the two first identical list propositions as a criterion for successful species identification and observed no incorrect results for both direct procedure from BCs and direct smear method without E/FA extraction from sub-cultures.

In this study, we used MALDI-TOF MS with tube-based E/FA extraction from sub-cultured colonies as a reference method for identification of yeasts. This method has been shown to be reliable in numerous studies and provided correct species identification in 94%−100% [20], [28], [32]–[34]. In line with these studies, this method yielded result in 100% (24/24) of isolates in our study. Thereof, 23 isolates achieved score ≥2.0 and one isolate of *C. dubliniensis* - score ≥1.7–<2.0. However, this reference method with E/FA extraction from sub-cultures on solid medium is time-consuming. Less laborious procedures would be clearly desirable for clinical lab routine. On the other hand, only 75% species identification rate with direct transfer of colony material to the target plate (direct smear method without protein extraction) in our study confirmed lower success rates with this easy-to-perform method as demonstrated in previous studies [33], [35]. Processing of the yeast isolates to extract fungal proteins helps to overcome the presence of a robust cell wall as an obstacle to the direct identification [30]. Based on several studies, on-plate extraction with adding of formic acid to the transferred colony material directly on the target plate might be a reasonable compromise between time expenditure and rate of yield in the routine clinical laboratory [29], [31], [36], [37].

We hypothesized that combined direct identification by MALDI-TOF MS and direct AFST by an automated system both from yeast cell pellets prepared with the same standard Sepsityper kit protocol can be a convenient method for routine diagnostics, provided that both deliver reliable results. While direct Vitek 2 inoculation from positive BCs has been intensively investigated for susceptibility testing of bacterial pathogens [38], [39], respective studies with yeasts are generally lacking. In the study of Machen *et al.*, same day identification plus antimicrobial susceptibility testing from positive BC bottles was possible by a combined lysis-filtration method with MALDI-TOF MS and the Vitek 2 system, however only 12 yeast-positive samples were included. For these samples, 100% agreement in susceptibility testing results was achieved [40]. Vitek 2 testing performed from isolated yeasts colonies has been shown to be a reliable AFST method in several studies [41]–[43], and was therefore used as a standard in our study.

The results of direct testing for amphotericin B, flucytosine and caspofungin were in 100% agreement with standard method, except of one mE for flucytosine in a *C. krusei* isolate. However, for both azole antifungals, fluconazole and voriconazole, the rate of VMEs was high –3/11 (27.3%) and 2/4 (50.0%), respectively (Table 3). All VMEs occurred with *C. albicans* isolates. Total VME, ME, mE rates for all antifungals amounted to 5/15 (33.3%), 1/54 (1.9%) and 1/70 (1.4%), respectively, with CA of 90% and EA of 88.6%. As required by both ISO [22] and FDA [23], we calculated above mentioned VME rates as divided by the number of resistant isolates determined by the standard method, and ME rates as divided by the number of susceptible isolates determined by the standard method, whereas mE rates are calculated as divided by the total number of tested isolates. If all error types are calculated related to the total number of isolates as denominator, VME, ME and mE rates amount to 3/14 (21.4%), 0% and 0% for fluconazole, and 2/14 (14.3%), 1/14 (7.1%) and 0% for voriconazole, respectively. This results in total VME, ME, mE rates for all antifungals 5/70 (7.1%), 1/70 (1.4%) and 1/70 (1.4%), respectively.

AFST result was generated for 16/22 (72.7%) of samples, while the testing of remaining samples was aborted. Most probably, this happened due to low inoculum and unsufficient growth of these samples. In addition, poor performance of direct AFST for azole antifungals can most probably be explained by low yeast cell numbers in the prepared pellets. Among 19 samples (S2 Table), for which both one pellet and two pellets were used for comparison, both CA (86.7% vs. 92.2%) and EA (84.4% vs. 90.2%) improved when two pellets inoculum was used. VME rate was lower with two pellets in this sub-group (45.5% vs. 27.3%), too.

Although time saving for AFST was remarkable with 15.1 h, we considered direct susceptibility testing using Sepsityper kit pellets to be of limited benefit - due to the fact that a considerable amount of measurements were aborted, and because the performance for azole antifungals was poor. The susceptibility of yeasts towards azoles is most variable among the antifungal drugs and correspondent testing has to be reliable. Future protocol developments by using increased inoculum, i.e. processing higher positive BC broth volume or the increased number of pellets, might give better results. Further studies would also benefit from inclusion more cases of different fungal species with different susceptibility patterns. In our study, number of samples was limited, particularly the number of positive BC bottles with *C. albicans* might have been underrepresented. This, however, corresponded to the detection rates in our laboratory.

In conclusion, direct MALDI-TOF MS using Sepsityper kit provides a reliable result for 62.5% of yeast-positive BCs. Direct Vitek 2 inoculation from Sepsityper pellet accelerated AFST for a part of samples, however, performance for azole antifungals is suboptimal. For yeasts, testing from subculture still remains indispensable to validate the direct finding.

## Supporting Information

S1 Figure **Protocol of MALDI-TOF MS procedure for direct identification from positive blood cultures using Sepsityper kit.**
(PDF)

S1 Table **Results of antifungal susceptibility testing by standard Vitek 2 method from 24 h sub-cultures, n = 23.**
(PDF)

S2 Table **Performance of direct antifungal susceptibility testing using inoculum from one yeast cell pellet compared to inoculum from two cell pellets.**
(PDF)
