# Supplementary material for: Rapid Identification and Susceptibility Testing of Candida spp. from Positive Blood Cultures by Combination of Direct MALDI-TOF Mass Spectrometry and Direct Inoculation of Vitek 2
Source: PLoS One. 2014 Dec 9;9(12):e114834. doi: 10.1371/journal.pone.0114834 (PMC4260948; doi:10.1371/journal.pone.0114834)
Supplement: S1 Figure — Protocol of MALDI-TOF MS procedure for direct identification from positive blood cultures using Sepsityper kit. (PDF) [file pone.0114834.s001.pdf]

Rapid identification and susceptibility testing of *Candida* spp. from positive blood cultures by combination of direct MALDI-TOF mass spectrometry and direct inoculation of Vitek 2

Evgeny A. Idelevich, Camilla M. Grunewald, Jörg Wüllenweber, Karsten Becker

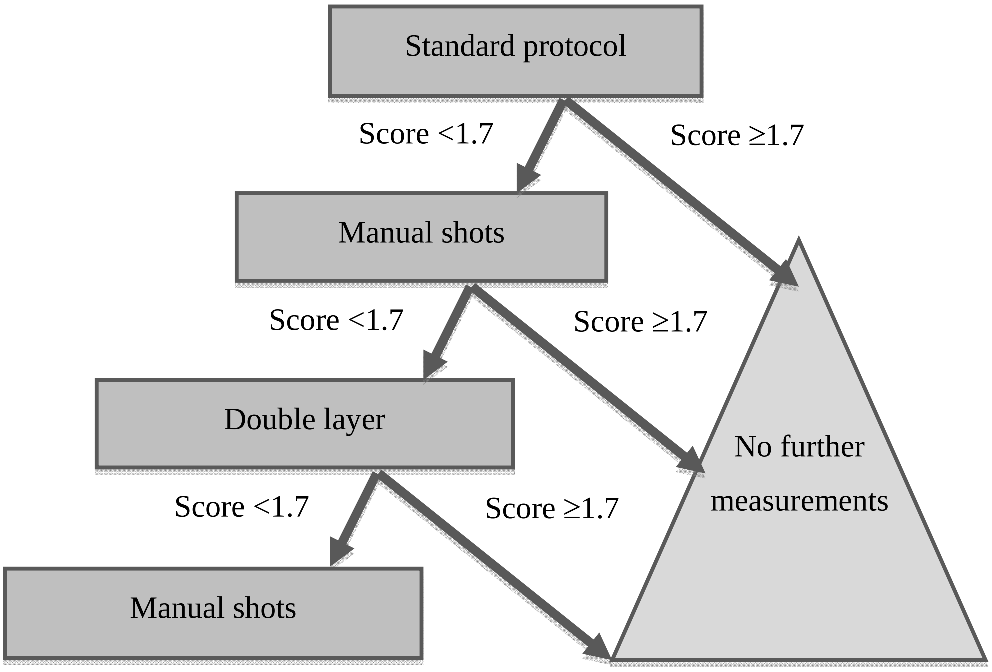

Figure S1. Protocol of MALDI-TOF MS procedure for direct identification from positive blood cultures using Sepsityper kit
