# Supplementary material for: Rapid Identification and Susceptibility Testing of Candida spp. from Positive Blood Cultures by Combination of Direct MALDI-TOF Mass Spectrometry and Direct Inoculation of Vitek 2
Source: PLoS One. 2014 Dec 9;9(12):e114834. doi: 10.1371/journal.pone.0114834 (PMC4260948; doi:10.1371/journal.pone.0114834)
Supplement: S1 Table — Results of antifungal susceptibility testing by standard Vitek 2 method from 24 h sub-cultures, n = 23. (PDF) [file pone.0114834.s002.pdf]

Rapid identification and susceptibility testing of *Candida* spp. from positive blood cultures by combination of direct MALDI-TOF mass spectrometry and direct inoculation of Vitek 2

Evgeny A. Idelevich, Camilla M. Grunewald, Jörg Wüllenweber, Karsten Becker

Table S1. Results of antifungal susceptibility testing by standard Vitek 2 method from 24 h sub-cultures, n=23.

Antifungal/ species	Number of isolates	Category			MIC[a], µg/ml	
		Susceptible	Intermediate	Resistant	50%	90%
**Amphotericin B**	**21**	**21**	**0**	**0**	**0.5**	**0.5**
C. albicans	9	9	0	0		
C. glabrata	11	11	0	0		
C. krusei	1	1	0	0		
**Fluconazole**	**23**	**7**	**1**	**15**	**4**	**8**
C. albicans	9	5	0	4		
C. glabrata	11	0	1	10		
C. dubliniensis	2	2	0	0		
C. krusei	1	0	0	1[b]		
**Voriconazole**	**23**	**15**	**0**	**8**	**≤0.12**	**4**
C. albicans	9	6	0	3		
C. glabrata	11	6	0	5		
C. dubliniensis	2	2	0	0		

C. krusei	1	1	0	0		
**Caspofungin**	**23**	**23**	**0**	**0**	**≤0.25**	**≤0.25**
C. albicans	9	9	0	0		
C. glabrata	11	11	0	0		
C. dubliniensis	2	2	0	0		
C. krusei	1	1	0	0		
**Flucytosine**	**23**	**22**	**1**	**0**	**≤1**	**≤1**
C. albicans	9	9	0	0		
C. glabrata	11	11	0	0		
C. dubliniensis	2	2	0	0		
C. krusei	1	0	1	0		

[a] MIC_{50} and MIC_{90} were not calculated for single species due to the low number of isolates

[b] intrinsic resistant
