# Supplementary material for: Rapid Identification and Susceptibility Testing of Candida spp. from Positive Blood Cultures by Combination of Direct MALDI-TOF Mass Spectrometry and Direct Inoculation of Vitek 2
Source: PLoS One. 2014 Dec 9;9(12):e114834. doi: 10.1371/journal.pone.0114834 (PMC4260948; doi:10.1371/journal.pone.0114834)
Supplement: S2 Table — Performance of direct antifungal susceptibility testing using inoculum from one yeast cell pellet compared to inoculum from two cell pellets. (PDF) [file pone.0114834.s003.pdf]

Rapid identification and susceptibility testing of *Candida* spp. from positive blood cultures by combination of direct MALDI-TOF mass spectrometry and direct inoculation of Vitek 2

Evgeny A. Idelevich, Camilla M. Grunewald, Jörg Wüllenweber, Karsten Becker

Table S2. Performance of direct antifungal susceptibility testing using inoculum from one yeast cell pellet compared to inoculum from two cell pellets.

Antifungal/ species	One pellet						Two pellets					
	IAC[a]	VME[b]	ME[b]	mE[b]	CA	EA	IAC[a]	VME[b]	ME[b]	mE[b]	CA	EA
**Amphotericin B**	**8**	**0**	**0**	**0**	**100%**	**100%**	**10**	**0**	**0**	**0**	**100%**	**100%**
C. albicans	3						4					
C. glabrata	5						6					
**Fluconazole**	**9**	**3 (37.5%)**	**0**	**0**	**66.7%**	**44.4%**	**10**	**2 (25.0%)**	**0**	**0**	**80%**	**60%**
C. albicans	4	3					4	2				
C. glabrata	5						6					
**Voriconazole**	**9**	**2 (66.7%)**	**1 (16.7%)**	**0**	**66.7%**	**77.8%**	**10**	**1 (33.3%)**	**1 (14.3%)**	**0**	**80%**	**90%**

C. albicans	4	2					4	1				
C. glabrata	5		1				6		1			
**Caspofungin**	**11**	**0**	**0**	**0**	**100%**	**100%**	**11**	**0**	**0**	**0**	**100%**	**100%**
C. albicans	6						5					
C. glabrata	5						6					
**Flucytosine**	**8**	**0**	**0**	**0**	**100%**	**100%**	**10**	**0**	**0**	**0**	**100%**	**100%**
C. albicans	3						4					
C. glabrata	5						6					
**TOTAL**	**45**	**5 (45.5%)**	**1 (2.9%)**	**0**	**86.7%**	**84.4%**	**51**	**3 (27.3)**	**1 (2.5%)**	**0**	**92.2%**	**90.2%**

[a] IAC, number of isolate-antifungal combinations

[b] Error rates are calculated according to ISO [22] and FDA [23] guidances. Very major errors (VME, %) - number of false susceptible results of direct AFST divided by the number of isolates tested resistant by the standard method, major errors (ME, %) - number of false resistant results of direct AFST divided by the number of susceptible isolates as determined by the standard method, minor errors (mE, %) - number of false categorizations involving intermediate result divided by the total number of tested isolates.
